# Fontan Circulation and Hepatic Venous Pressure Gradient: Are Bridging Collaterals One of the Weak Links?

**DOI:** 10.1016/j.cjcpc.2025.01.005

**Published:** 2025-02-07

**Authors:** Ashish H. Shah, Katrijn Jansen, Alexander C. Egbe, Iain D.C. Kirkpatrick

**Affiliations:** a Section of Cardiology, Department of Internal Medicine, Max Rady Faculty of Health Sciences, University of Manitoba, Winnipeg, Manitoba, Canada; b Department of Cardiology, Freeman Hospital, Newcastle-Upon-Tyne, United Kingdom; c Department of Cardiovascular Medicine, Mayo Clinic, Rochester, Minnesota, USA; d Department of Radiology, Max Rady Faculty of Health Sciences, University of Manitoba, Winnipeg, Manitoba, Canada

Fontan-associated liver disease ranges from hepatic congestion to cirrhosis, and more advanced hepatic pathology is associated with increased mortality.^1^ There are emerging discussions around the role of heart-alone vs heart-liver transplantation in treating patients with failing Fontan circulation, but the precise indications supporting one approach over another remain unclear. In the absence of precise objective indications, some of the parameters used in patients with non–Fontan-related liver pathology have been incorporated in assessing Fontan patients. The hepatic venous pressure gradient (HVPG) is one such parameter that represents the difference between the portal and hepatic venous pressure. HVPG is usually elevated in patients with cirrhosis; normally HVPG is ≤5 mm Hg, whereas ≥5 mm Hg defines portal hypertension.^2^^,^^3^ Among patients with cirrhosis (non-Fontan), an HVPG of ≥12 mm Hg identifies those at a higher risk of adverse outcomes. Although the HVPG measurement is crucial for risk stratification and treatment selection in non-Fontan patients, the values are typically within a normal range among patients with Fontan-associated liver disease, and its correlation with liver fibrosis and the impact on outcomes in this subgroup remain unclear.^2^^,^^4^ Studies evaluating hemodynamics in Fontan patients have demonstrated HVPG to typically be within a normal range (0-3 mm Hg).^4^^,^^5^ In contrast to the published data for non-Fontan patients, HVPG did not have any correlation with sinusoidal or portal fibrosis, nor transplant-free survival.^4^

At a single institution, 15 of 62 Fontan patients were investigated by cardiac catheterization at the discretion of their physicians, of whom 5 were assessed for HVPGs. Nonocclusive angiogram demonstrated a normal appearing hepatic vein (Video 1 , view video online). The observed HPVG was 0-2 mm Hg in all 5, but 2 of the 5 patients were noted to have bridging collaterals connecting the distal hepatic vein (beyond the point of balloon occlusion) to the inferior vena cava, equalizing the pressure; hence, no gradient was noted despite the elevated Fontan pressure (Fig. 1; Video 2 , view video online). Interestingly, angiogram in the inferior vena cava also demonstrated filling of these bridging collaterals (Fig. 1; Video 3 , view video online). None of these patients were investigated by liver biopsy at the same time.

**Figure 1:**
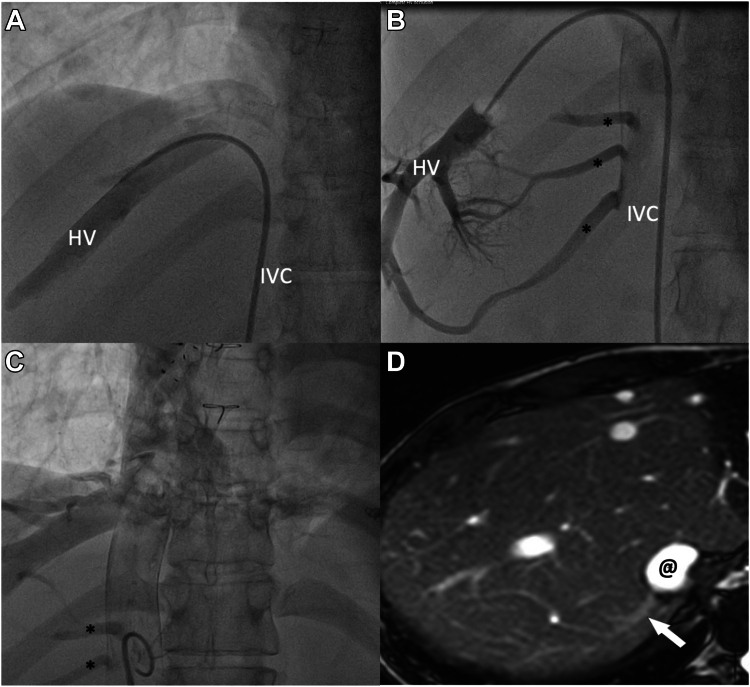
Hepatic venovenous collaterals. (**A**) Nonocclusive hepatic venous angiography, (**B**) occlusive hepatic venous angiography demonstrating bridging collaterals or accessary hepatic veins (∗) con necting the inferior vena cava, (**C**) inferior vena cava angiogram demonstrating these accessary hepatic veins, and (**D**) transverse b-SSFP MR image demonstrating one of the accessory hepatic veins (**arrow**) draining into the inferior vena cava (@) in segment VII. b-SSFP MR, balanced steady-state free precession magnetic resonance; HV, hepatic vein; IVC, inferior vena cava.

Normally described hepatic venous anatomy includes right, middle, and left hepatic veins. One of the most commonly observed anatomic variations is an accessory right hepatic vein (single or multiple) draining portions of the right lobe (typically from segment VI or VII) directly into the inferior vena cava. An accessary inferior hepatic vein may drain approximately 10% of the total hepatic venous flow. It is important to define hepatic venous drainage in the setting of liver surgery or transplantation. However, we have not come across any publication describing this anomaly as it may relate to the investigation of Fontan patients. Although our sample size is small, such drainage would not allow one to measure HVPG. Moreover, unless carefully injected into the hepatic vein beyond the point of occlusion, one may not be aware of such an anomaly. The treating physician should be cognizant of the possibility of such an anomaly, as conventionally measured HVPG may not be reliable.

**Novel Teaching Points**

• Despite progressive liver fibrosis, Fontan patients generally do not exhibit elevated hepatic venous pressure gradients.
• Some Fontan patients may have bridging collaterals or accessory hepatic veins that connect to the inferior vena cava, equalizing pressures between the distal and proximal hepatic veins. In such patients, wedge pressure should be measured from an alternative vein, including the left hepatic vein, that does not exhibit abnormal communication.
• Hepatic venous angiography is essential to identify these anomalies and ensure accurate hepatic venous pressure gradient measurement.

## Supplementary Material

Video 1 Nonocclusive hepatic venogram.

Video 2 Hepatic venogram with balloon occlusion.

Video 3 Inferior vena cava angiogram.
